# Effects of different reminder strategies on first-time mammography screening among women in Taiwan

**DOI:** 10.1186/s12913-020-4948-6

**Published:** 2020-02-12

**Authors:** Miao-Ling Lin, Joh-Jong Huang, Shu-Hua Li, Fang-Hsin Lee, Ming-Feng Hou, Hsiu-Hung Wang

**Affiliations:** 1 Long-Term Care Division, Department of Health, Kaohsiung City Government, No. 2, Sihwei 3rd Road, Lingya District, Kaohsiung City, 80203 Taiwan; 2 College of Nursing, Kaohsiung Medical University, No. 2, Sihwei 3rd Road, Lingya District, Kaohsiung City, 80203 Taiwan; 3 Graduate Institute of Gender Studies, Kaohsiung Medical University, No. 100 Shih-Chuan 1st Road, San-Ming District, Kaohsiung, 80708 Taiwan; 4 Department of Nursing, Chung Hwa University of Medical Technology, No. 89, Wenhua 1st St., Rende District, Tainan, 71703 Taiwan; 5 Graduate Institute of Clinical Medicine, Kaohsiung Medical University, No. 100 Shih-Chuan 1st Road, San-Ming District, Kaohsiung, 80708 Taiwan; 6 College of Nursing, Kaohsiung Medical University, No. 100, Shih-Chuan 1st Road, Kaohsiung, 807 Taiwan

**Keywords:** Mammography screening, Middle-aged women, Reminder interventions, Taiwan

## Abstract

**Background:**

The study’s purpose was to examine the effectiveness of different reminder strategies on first-time free mammography screening among middle-aged women in Taiwan.

**Methods:**

A quasi-experimental design with random assignment was adopted to divide the participants into three Reminder Strategies groups (mail reminder, telephone reminder, and combined mail and telephone reminders) and one control group. This study recruited 240 eligible middle-aged women, and 205 of them completed the study. Upon the completion of data collection, mail reminders were provided to women of the first group; telephone reminders were provided to the second group; mail followed by telephone reminders were provided to the third group, and the usual postcards were provided to the control group 1 month after the interventions. Two follow-up assessments were conducted 1 and 3 months after the intervention to collect mammography-screening behaviors from all groups.

**Results:**

The findings showed that, compared to the control group, more participants in the intervention groups underwent mammography screening after receiving reminder interventions. Telephone contact as reminder was found to have the most significant influence among the interventions (OR = 5.0556; 95% CI = 2.0422–13.5722).

**Conclusions:**

Government and healthcare providers are recommended to consider adopting the telephone reminder strategy to encourage women to undergo their first-time mammography screening.

## Background

In Taiwan, breast cancer is a leading cause of disease and has the fourth highest mortality rate among cancers [1]. The American Cancer Society (ACS) has pointed out that the mammography is the most effective tool for detecting breast cancer [2]. Even though there is some controversy over the harm of a mammography to women such as over-diagnosis and over-treatment [3], a mammography is still the most recommended tool for screening, diagnostic assessment, and high-risk monitoring of women without breast cancer symptoms [4]. According to the statistics from the National Health Administration of Taiwan in 2014, the incidence rate of breast cancer was the highest among females aged 45–49 [5]. These statistics triggered much discussion as to whether government should provide free mammograms for younger women under the age of 50. Although mammography screening has been shown to be an effective strategy for early detection of breast cancer, 63.3% of Taiwanese women aged 45–69 have never received mammography screening [6]. The reasons why Taiwanese women do not undergo mammography screening are reported as “no time,” “forgetfulness,” “too cumbersome,” and “laziness,” followed by the perception of no need [7]; therefore, emphasizing the importance of effectively designing, planning, and implementing comprehensive interventions to raise breast cancer awareness and promote mammography screening are both necessary to optimize health outcomes for these targeted women. In Taiwan, the government has offered free mammography screening to women aged between 45 and 69 every 2 years. However, compared with other age groups, the mammography screening rate among women aged 45–49 is the lowest [5]. A previous study reported that Asian women in their 40s appeared to receive more benefits from mammography screening compared to age-matched non-Asian women in the US [8]. A primary care visit within a year and an increased number of primary care visits within a year were the factors associated with screening initiation [9]. Healthcare providers could play critical roles in influencing female’s mammography screening behaviors; therefore, encouraging middle-aged women to undergo mammography screening for early detection of breast cancer should be considered by healthcare providers.

Many strategies are often used to increase women’s mammography screening rates, such as personalized reminders, public health messages, individual education, elimination of mobility barriers, and group education [10]. A study in Taiwan pointed out that the use of tailored health education websites could effectively increase women’s positive perceptions of mammography and significantly raise their intention to obtain a mammogram [11]. In addition, using low-cost reminder strategies to increase the mammogram rate is an important part of early breast cancer prevention [12]. A previous study noted that the combination of mail and telephone interventions substantially increased women’s mammography screening rates, because a telephone intervention can reduce perceived barriers to action, enhance perceived benefits of action, and increase the likelihood of action [13].

In Taiwan, the government has lowered the age criteria for receiving mammography examination from 55 to 45 years considering the increasing incidence rate of breast cancer among younger women, so assisting women aged above 45 to undergo mammography examinations as soon as possible has become an important community nursing responsibility [6]. In Taiwan, almost every household has a telephone or mobile phone, and as postal services are also very convenient, both telephone calls and mail transmissions make communication easier. A previous study showed that employing appropriate reminder strategies to assist women in arranging their first mammography examination increased their 18-month follow-up examination rates by up to 68% [14]. Breast cancer is a health threat to Taiwanese women. The government provides mammography to women over the age of 45, which is expected to achieve early detection of symptoms and early treatment, so improvement of screening rates is extremely important. Identification of effective reminder strategies for 45-year-old women to receive their first free mammography screening service from the government will help promote breast cancer prevention and enable further treatment measures from community health nurses in Taiwan. Many of previous empirical researches confirmed the effectiveness of the reminder strategy. This study will explore reminder strategies that work for women who were first screened in Taiwan.

## Methods

### Study design

A quasi-experimental design with random assignment was adopted to divide the participants into four groups, including three intervention groups (mail reminder, telephone reminder, combined mail and telephone reminder) and a control group, to examine the impact of different reminder strategies. The study recruited women who had just turned 45 years of age because they were legitimately entitled to receive free mammography screening funded by the Taiwan government. Aside from age, the recruiting criteria were also limited to those women who (1) had never received free mammography examination; (2) had never received a mastectomy; (3) lived in Kaohsiung city; and (4) were willing to participate in the study. The study was conducted from March 2015 to March 2016 in Kaohsiung City, Taiwan. The research was approved by the Institutional Review Board (IRB No: KSPH-2014-35) before conducting the study.

### Participants

This study adopted a quasi-experimental design; participants were randomly divided into four groups. The sample size of the Binary Logistic Regression study with G-power 3.1.9.2 was estimate with the parameters in the literature as a reference, with α being set as 0.05 and power being set as 0.80. Two hundred participants were required with each group of 50 participants. Besides, an extra 20% of the sample was required in case of missing answers, and thus, 240 women were recruited in total. Participants were recruited from Kaohsiung City of Taiwan, with the researchers randomly selecting participants from the National Cancer Registry system. The National Cancer Registry system records the cancer screening records and results of virtually all Taiwanese who have undergone screening, and documents and lists all women that are eligible for mammography screening. The participants must have just turned 45 and have no mammography records at all. Women who qualify for mammography screening are recorded in this system. All participating women are numbered after they are invited and agreed to participate in the study.

Two hundred and forty women were invited to join this study and were randomly assigned into four groups including the mail reminder, telephone reminder, combined mail and telephone reminder, and control groups. Sixty participants were assigned to each group, with demographic data being collected before the intervention. Aside from those who did not complete the study, 205 women completed the whole study process, including 46 in the mail reminder group, 48 in the telephone reminder group, 51 in the combined mail and telephone reminder group, and 60 in the control group.

### Intervention strategies

**Fig. 1:**
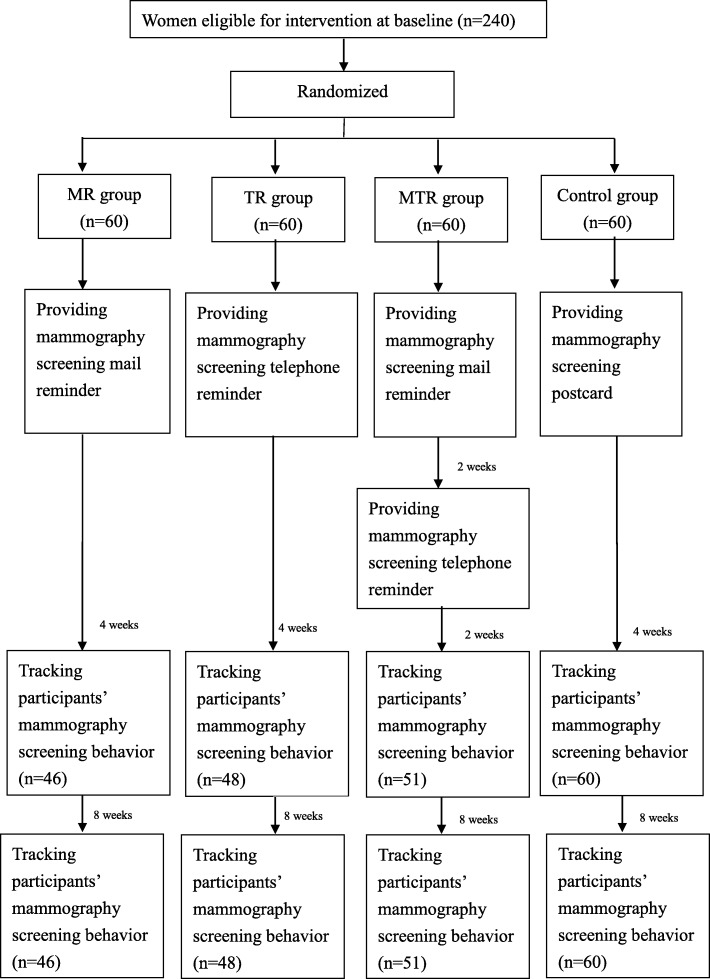
The flow chart of the study. Conceptual framework of the study mammography screening implementation of the four groups’ participants before and after 4 weeks and 12 weeks reminder intervention

Participants were randomly assigned to four groups, including three intervention groups (mail reminder, telephone reminder, combined mail and telephone reminder) and one control group. All participants were provided with reminder strategies after their agreement to participate in the study and they completed basic information, while women in the control group also completed basic information. In the mail reminder group (MR group), the mailed information was checked by the experts on public health and breast cancer to ensure that it was adequate and proper. In the telephone reminder group (TR group), the participants were phoned by public healthcare providers and given the same information as the MR group. Researchers completed training according to the instruction manual before they start calling participants to ensure that their expressions were consistent. During the study, participants in the telephone group (TR group) could be assisted by health care providers to schedule a mammography screening if needed. In the combined mail and telephone reminders group (MTR group), the participants first received a mail with the same information and then received a telephone reminder 2 weeks after receiving the mail. The participants of MTR also had the opportunity to schedule a mammography screening. No information in any format would be given to the control group at the beginning of the study. The usual care provided via postcard with free mammography screening information was given to the control group by the public healthcare center within 1 month after they had turned 45 years of age. No participant has scheduled an appointment before receiving any invitation. The flow chart of the study is shown in Fig. 1.

### Measures and analysis

Before the interventions, the demographic data of the participants for each group were collected. The dependent variable of this study was the participants’ mammography screening behavior, which was checked through examination records in the Taiwan National Cancer Registry System. Confirming mammographic screening behavior from the National Cancer Registry System can reduce self-reported errors. The data of TR, MR and MTR groups were checked 1 month and 3 months after the intervention, while participants in the control group received postcards at different times; therefore, 1 month and 3 months after the intervention, the data of the control group were collected. Descriptive statistics were used to analyze the demographic characteristics of the participants, while Analysis of Variance (ANOVA) was used to analyze the effectiveness of mammography screening rate among different interventions and Chi-square tests were used to analyze the differences of the mammography screening rate after the interventions provided at different times; additionally, a logistic regression analysis was adopted to explore the relationship between different mammography reminders and mammography screening behaviors.

## Results

In total, 205 women participated in the study. The participants’ demographic characteristics are shown in Table 1. No significant differences were found among the four groups in the basic demographic variables that were considered confounding variables based on the literature and were controlled for further analysis [15–18].

**Table 1:** Participants’ demographic characteristics (*n* = 205)

Variable	MR	TR	MTR	C	total	*p*-value of chi-square
	*N(%)*	*N(%)*	*N(%)*	*N(%)*		
Educational level						0.9684
High school and below	22 (48.3)	24 (50.0)	23 (45.1)	28 (46.7)	97 (47.3)	
College and above	24 (51.7)	24 (50.0)	28 (54.9)	32 (53.3)	108 (52.7)	
Marital status						0.2533
Unmarried	4 (8.7)	9 (19.8)	6 (11.8)	11 (18.3)	30 (14.6)	
Married	37 (80.4)	37 (77.1)	38 (74.5)	43 (71.7)	155 (75.6)	
Divorced/separated/widowed	5 (10.9)	2 (4.1)	7 (13.7)	6 (10.0)	20 (9.8)	
Employment status						0.9697
Unemployed	4 (8.7)	8 (16.7)	11 (21.6)	9 (15.0)	32 (15.6)	
Employed with full-time job	40 (87.0)	34 (70.8)	32 (62.7)	47 (78.3)	153 (74.6)	
Employed with part-time job	2 (4.3)	6 (12.5)	8 (15.7)	4 (6.7)	20 (9.8)	
Personal monthly income (Taiwanese dollars)						0.1536
Below 30,000	22 (47.8)	30 (62.5)	29 (56.9)	30 (50.0)	111 (54.1)	
30,000–50,000	19 (41.3)	8 (16.7)	15 (29.4)	16 (26.7)	58 (28.3)	
Above 50,000	5 (10.9)	10 (20.8)	7 (13.7)	14 (23.3)	36 (17.6)	
Perception of the income						0.4660
Insufficient	14 (30.4)	18 (37.5)	21 (41.2)	14 (23.4)	67 (32.6)	
Balanced	16 (34.8)	13 (27.1)	17 (33.3)	23 (38.3)	69 (33.7)	
Sufficient	16 (34.8)	17 (35.4)	13 (25.5)	23 (38.3)	69 (33.7)	
Medical insurance other than National Health Insurance						0.6332
No	4 (8.7)	6 (12.5)	3 (5.9)	4 (6.7)	17 (8.3)	
Yes	42 (91.3)	42 (87.5)	48 (94.1)	56 (93.3)	188 (91.7)	
Cancer history						0.5330
No	46 (100.0)	46 (95.8)	49 (96.1)	57 (95.0)	198 (96.6)	
Yes	0 (0.0)	2 (4.2)	2 (3.9)	3 (5.0)	7 (3.4)	
Family cancer history						0.9826
No	39 (84.8)	40 (83.3)	44 (86.3)	51 (85.0)	174 (84.9)	
Yes	7 (15.2)	8 (16.7)	7 (13.7)	9 (15.0)	31 (15.1)	
Delivery history						0.2410
No	5 (10.9)	11 (22.9)	8 (15.7)	15 (25.0)	39 (19.0)	
Yes	41 (89.1)	37 (77.1)	43 (84.3)	45 (75.0)	166 (81.0)	

1. Demographic variables with a small sample size were compared with nonparametric statistics

2. *MR* Mail reminder, *TR* Telephone reminder, *MTR* Combined mail and telephone reminder, *C* Control group

Three months after the intervention, the TR group showed the highest screening rate, where 21 women (43.8%) received screening. The screening rate in the control group was the lowest, where only eight women (13.3%) underwent screening. The differences among the four groups were significant (*p* = 0.0042). As shown in Table 2, there was a significant increase in screening rates of the three intervention groups, both from 1 month to 3 months after the interventions (*p* <  0.0001) for all three groups.

**Table 2:** Differences in mammography screening rates between participants at 1 month and 3 months after interventions

	One month after intervention		Three months after intervention		*p*-value of chi-square
	Mammography N (%)	No Mammography N (%)	Mammography N (%)	No Mammography N (%)	
MR group	9 (19.6)	37 (80.4)	16 (34.8%)	30 (65.2%)	< 0.0001
TR group	11 (22.9)	37 (77.1)	21 (43.7%)	27 (56.3%)	< 0.0001
MTR group	8 (15.7)	43 (84.3)	18 (35.3)	33 (64.7%)	< 0.0001

*MR* Mail reminder, *TR* Telephone reminder, *MTR* Combined mail and telephone reminders

Logistic regression with the control group serving as the reference group was performed to determine if mammography screenings had differences between the MR, TR and MTR groups during a follow-up period of 3 months. Age, education, marital status, income, employment status, medical insurance, history of cancer and delivery experience were adjusted variables by implementing multiple logistic regression. The results are presented in Table 3. Three months after the interventions, the odds ratio of receiving MR intervention on mammography screening was 3.47 (95% CI = 1.36–9.46, *p* = 0.0112); indicating that the likelihood of women who received an MR to undergo screening was 3.47-fold greater than in the control group. The odds ratio of receiving TR intervention on screening was 5.06 (95% CI = 2.04–13.57, *p* = 0.0004); indicating that the likelihood of women who received a TR to undergo screening was 5.06-fold greater than those in the control group. The odds ratio of receiving the MTR intervention on screening was 3.55 (95% CI = 1.42–9.51, *p* = 0.0083); indicating that the likelihood of women who received an MTR to undergo mammography screening was 3.55-fold greater than those in the control group.

**Table 3:** Odds ratio of mammography screening of different reminder strategies by the end of the study (*n* = 205)

	Odds Ratio	95% CI		Significance (*p*-value)
		Lower	Upper	
Three months after intervention				
MR group v.s. C group	3.47	1.36	9.46	0.0112
TR group v.s. C group	5.06	2.04	13.57	0.0007
MTR group v.s. C group	3.55	1.42	9.51	0.0083

*MR* Mail reminder, *TR* Telephone reminder, *MTR* Combined mail and telephone reminders, *C* Control, *CI* Confidence interval

## Discussion

The main findings of this study showed that, compared to the control group, more participants in the three intervention groups (TR, MR, MTR) underwent mammography screening after receiving different reminder interventions. Telephone contact as reminder was found to have the most significant influence among the interventions.

A previous study found that poor access to health information for screening was significantly associated with being both overdue and never screeners [19]. In the past, public health centers in Taiwan usually used telephone calls, letters, and other reminder strategies to assist women to take the examination. However, the effectiveness of various strategies for the target population has not been supported by empirical research.

This study showed that the effects of TR, MR, and MTR reminders of a free mammography examination for 45-year-old women were all more effective than postcard invitations; in fact, the proportion of telephone reminder participants who underwent screening was 43.7%. This was higher than MR, MTR and postcard strategies and also higher than the findings of the survey conducted by the Health Promotion Administration of Taiwan in the same year, which concluded that the proportion of women aged 45–49 years who had undergone free biennial mammography screening was 34.3% [1].

Past research indicated that the content of the mail reminders, such as procedures of mammography screening, risks, and benefits, had no detectable impact on women’s participation in mammography examinations; however, a clear mammography screening invitation could increase screening rates in women [17]. In this study, the postcard that women received in the control group only held information concerning the free examination, and there was a lack of information assisting participants in the how-to of mammography screening appointments; therefore, the mammography screening rate of the control group was lower than that of the MR group. This also showed that, in encouraging women to take mammograms by mail reminders, placing information to help arrange the examinations such as setting an appointment time is useful. Previous studies have revealed that, compared to a mail reminder alone, a mail reminder combined with a telephone reminder could increase the chances of women undergoing a mammography screening 2.2-fold; however, a mail with an automated telephone message reminder was not more effective than a mail reminder alone in enhancing screening rates [20, 21]. In this study, the mammography screening rate of the MTR group was not significantly higher than the TR group. Previous studies revealed that women who were more responsive to the first reminder of mammography screening were less influenced by the following reminders [22]. This implied that women might have already decided to undergo the mammography screening once receiving the mail reminder, so the telephone reminder boost within 2 weeks might not change their original examination intent.

In this study, in the MTR group, the time between sending letters and call time was 2 weeks. Perhaps the interval needs to be reconsidered. The tracking results of the MTR group after 3 months were similar to those of the MR group. Perhaps the two-week interval design had limited difference among the results of MTR and MR groups. In future studies, exploring shortening the prompt interval or extending the tracking time is suggested in order to further confirm the necessity of using mail and telephone reminders together. As a result of the study, the screening rate of mammography among women in the control group was only 13%, which was lower than the findings of the survey conducted by the Health Promotion Administration of Taiwan in the same year [5]. The fact that women in the control group did not receive any form of reminder strategy could be one of the reasons. The mammography screening rate of women in the same year by the Health Promotion Administration of Taiwan was influenced by various kinds of education, reminder strategies and experience. The most critical reason for the difference is that our study followed up the screening rate 3 months after the intervention, whereas the Health Promotion Administration of Taiwan followed up the screening rate for the period of 2 years. It is suggested that any future study should follow up the long-term effect of the interventions.

The study showed that participating in mammography screening was significantly higher for women contacted by telephone than through routine advocacy [23]. A study found that patient navigators could increase mammography adherence in previously non-adherent women by making the screening appointment while the woman was on the phone [24]. The percentage of women who received mammography screening was 4% higher among those who received volunteer phone calls than those in usual care. Importantly, the phone content must cover the individual mammography history, mammography appointment date, and type of mammography facility in the area [25]. The telephone intervention could reduce individuals’ perceived mobility barriers, increase their perceived behavioral benefits, and the likelihood of taking action [13, 26]; this could provide the participants with the quickest channel for making a mammography screening appointment, which might facilitate their willingness to undergo the examination. A telephone call, in addition to providing a reminder about mammography examinations, also provides immediate inspection information and interaction with participants more directly. These are all important factors for the telephone reminder to effectively increase the rate of the first-time mammography screening for 45-year-old women. It is suggested that a cost-effective analysis can be conducted in a future study to confirm the suitability of the telephone reminder strategy as the priority choice for women with first-time free mammography screening.

A previous study found that employing appropriate reminder strategies to assist women to arrange their first mammography examination would increase their 18-month follow-up examination rates by up to 68% [14]. Therefore, the use of telephone reminder strategies to assist 45-year-old women to receive the first-time free mammography screening from the government will not only transmit screening information, but also directly assist in arranging such screening. This will not only help improve first mammography screening rate but also help improve follow-up screening rate in women. The study showed that the telephone reminder was the more effective strategy for 45-year-old women undergoing mammography for the first time. Healthcare providers and policy makers are recommended to consider adopting the telephone reminder strategy to encourage 45-year-old women to undergo their first-time mammography screening. It is also suggested that clinicians consider investigating the impact of telephone reminder on women of different ages and mammography experience. Community health nurses can train community volunteers to assist with the implementation of mammography reminder services and increase manpower resources for promoting this activity. This will help increase women’s first-time mammography screening rates. In future studies, we recommend conducting cost-analysis research.

### Study limitations

This sample is for women aged 45 years, and the results of the study being extrapolated to women of other ages might be limited. The study excluded women with a history of breast cancer and who had undergone mastectomy, so the external validity of the study is limited. In addition, the study tracked mammography screening rates of the targeted women after 3 months of interventions and might have missed the screening data of those women that had been scheduled for a longer period of time.

## Conclusions

Providing mammography reminders can increase the percentage of women receiving their first-time mammogram. Telephone reminder, mail reminder, and combined mail and telephone reminder strategies are all more effective than regular postcards. Government and healthcare providers could choose appropriate strategies based on the status of manpower and funds. This study provides proof of the principle that warrants further investigation in a larger trial, although it does not offer sufficiently robust evidence upon which to advise a policy change.

## Data Availability

Data cannot be made publically available owing to the fact that the privacy of individual participants cannot be compromised. However, the dataset is available from the corresponding author on reasonable request.

## Abbreviations

C: Control group
CI: Confidence interval
MR: Mail reminder
MTR: Combined mail and telephone reminder
OR: Odds ratio
TR: Telephone reminder

## References

[1] Cancer Registry Annual Report, 2014. Available from: https://www.hpa.gov.tw/Pages/Detail.aspx?nodeid=269&pid=7330. Accessed 19 Dec 2019.

[2] American Cancer Society (n. d.). Breast cancer facts & figures 2007–2008. Available from: www.cancer.org/downloads/STT/BCFF-Final.pdf. Accessed 19 Dec 2019.

[3] Bjørndal A, Forsetlund L (2007). Mammography screening of women 40–49. NIPH.

[4] Hanson K, Montgomery P, Bakker D, Conlon M (2009). Factors influencing mammography participation in Canada: an integrative review of the literature. Curr Oncol.

[5] Statistical Yearbook of Health Promotion 2014 Taiwan. Available from: https://www.hpa.gov.tw/Pages/Detail.aspx?nodeid=268&pid=7529. Accessed 19 Dec 2019.

[6] Statistical yearbook of health promotion. Available from: https://www.hpa.gov.tw/Pages/Detail.aspx?nodeid=268&pid=7529. Accessed 19 Dec 2019.

[7] Wu TY, Chung S, Yeh MC, Chang SC, Hsieh HF, Ha SJ (2012). Understanding breast Cancer screening practices in Taiwan: a country with universal health care. APOCP..

[8] Tsuchida J, Nagahashi M, Rashid OM, Takabe K, Wakai T (2015). At what age should screening mammography be recommended for Asian women?. Cancer Med.

[9] Beaber EF, Tosteson ANA, Haas JS, Onega T, Sprague BL, Weaver DL (2017). Breast cancer screening initiation after turning 40 years of age within the PROSPR consortium. PMC.

[10] Lobb R, Opdyke KM, McDonnell CJ, Pagaduan MG, Hurlbert M, Gates-Ferris (2011). Use of evidence-based strategies to promote mammography among medically underserved women. Am J Prev Med.

[11] Lin, Effken (2010). Effects of a tailored web-based educational intervention on women's perceptions of and intentions to obtain mammography. J Clin Nurs.

[12] Phillips L, Hendren S, Humiston S, Winters P, Fiscella K (2015). Improving breast and colon cancer screening rates: a comparison of letters, automated phone calls, or both. JABFM..

[13] Hegenscheid K, Hoffmann W, Fochler S, Domin M, Weiss S, Hartmann B (2011). Telephone counseling and attendance in a national mammography-screening program a randomized controlled trial. Am J Prev Med.

[14] Tang TS, Patterson SK, Roubidoux MA, Duan L (2009). Women’s mammography experience and its impact on screening adherence. Psychooncology..

[15] Calo WA, Vernon SW, Lairson DR, Linder SH (2016). Area-level socioeconomic inequalities in the use of mammography screening: a multilevel analysis of the health of Houston survey. Women Health Issuses.

[16] Carney PA, O'Malley JP, Gough A, Buckley DI, Wallance J, Fagnan LJ (2013). Association between documented family history of cancer and screening for breast and colorectal cancer. Prev Med.

[17] Giordano L, Stefanini V, Senore C, Frigerio A, Castagno R, Marra V (2011). The impact of different communication and organizational strategies on mammography screening uptake in women aged 40-45 years. Eur J Publ Health.

[18] Weber MF, Cunich M, Smith DP, Salkeld G, Sitas F, O'Connell D (2013). Sociodemographic and health-related predictors of self-reported mammogram, faecal occult blood test and prostate specific antigen test use in a large Australian study. BMC Public Health.

[19] Shields M, Wilkins K (2009). An update on mammography use in Canada. Health Rep.

[20] Arcas MM, Buron A, Ramis O, Esturi M, Hernandez C, Macia F (2014). Can a mobile phone short message increase participation in breast cancer screening programmes. Rev Calid Asist.

[21] Fortuna RJ, Idris A, Winters P, Humiston SG, Scofield S, Hendren S (2013). Get screened: a randomized trial of the incremental benefits of reminders, recall, and outreach on cancer screening. JGIM.

[22] Costanza ME, Luckmann R, White MJ, Rosal MC, Cranos C, Reed G (2011). Design and methods for a randomized clinical trial comparing three outreach efforts to improve screening mammography adherence. BMC Health Serv Res.

[23] Barr JK, Franks AL, Lee NC, Antonucci DM, Rifkind S, Schachter M (2001). A randomized intervention to improve ongoing participation in mammography. Am J Manag Care.

[24] Payton CA, Sarfaty M, Beckett S, Campos C, Hilbert K (2015). Does telephone scheduling assistance increase mammography screening adherence?. Am J Manag Care.

[25] Goelen G, De Clercq G, Hanssens S (2010). A community peer-volunteer telephone reminder call to increase breast cancer-screening attendance. Oncol Nurs Forum.

[26] Baysal HY, Gozum S (2011). Effects of health beliefs about mammography and breast cancer and telephone reminders on re-screening in Turkey. Asian Pac J Cancer Prev.

